# Band Gap Implications on Nano-TiO_2_ Surface Modification with Ascorbic Acid for Visible Light-Active Polypropylene Coated Photocatalyst

**DOI:** 10.3390/nano8080599

**Published:** 2018-08-07

**Authors:** Chiara Anna D’Amato, Rita Giovannetti, Marco Zannotti, Elena Rommozzi, Marco Minicucci, Roberto Gunnella, Andrea Di Cicco

**Affiliations:** 1 School of Science and Technology, Chemistry Division, University of Camerino, 62032 Camerino, Italy; elena.rommozzi@unicam.it; 2 School of Science and Technology, Physics Division, University of Camerino, 62032 Camerino, Italy; marco.minicucci@unicam.it (M.M.); roberto.gunnella@unicam.it (R.G.); andrea.dicicco@unicam.it (A.D.C.)

**Keywords:** heterogeneous photocatalysis, TiO_2_, ascorbic acid, surface modification, band gap energy, Alizarin Red S

## Abstract

The effect of surface modification using ascorbic acid as a surface modifier of nano-TiO_2_ heterogeneous photocatalyst was studied. The preparation of supported photocatalyst was made by a specific paste containing ascorbic acid modified TiO_2_ nanoparticles used to cover Polypropylene as a support material. The obtained heterogeneous photocatalyst was thoroughly characterized (scanning electron microscope (SEM), RAMAN, X-ray diffraction (XRD), X-ray photoelectron spectroscopy (XPS), photoluminescence (PL), and Diffuse Reflectance Spectra (DRS) and successfully applied in the visible light photodegradation of Alizarin Red S in water solutions. In particular, this new supported TiO_2_ photocatalyst showed a change in the adsorption mechanism of dye with respect to that of only TiO_2_ due to the surface properties. In addition, an improvement of photocatalytic performances in the visible light photodegration was obtained, showing a strict correlation between efficiency and energy band gap values, evidencing the favorable surface modification of TiO_2_ nanoparticles.

## 1. Introduction

In recent years, progress in industrialized society has caused serious environmental problems due, for example, to the discharge of a wide variety of environmental contaminants from residential, commercial, and industrial sources [1]. Azo-dyes and contaminants released from the textile industry are mostly non-biodegradable pollutants, toxic, and also resistant to degradation using the traditional treatment methods. For these reasons they represent an important source of environmental contamination. Color removal from wastewater is an important issue because only small amounts of dyes present high effects both on the color and water quality. Consequently, it is necessary to find an effective method of wastewater treatment to remove dye pollutants and their colors from textile effluents [2]. Nowadays, a greater challenge in the environmental field relies on the treatment of contaminants and advanced oxidation processes (AOPs) have been considered as alternatives to traditional water treatment technologies [1,3]. Among several AOPs, TiO_2_ based photocatalysis has received huge attention as one of the most viable environmental clean-up technologies [1]; TiO_2_ has been considered as among the most promising materials due to its high chemical stability, low cost, chemical inertness, commercial availability, and outstanding photocatalytic activity [4,5,6,7]. TiO_2_ has been extensively used in many industrially relevant processes ranging from environmental applications to clean energy, from paints to cosmetics and medicine [7]. The semiconductor materials find application in medicine as a photosensitizer for photodynamic and photothermal therapy of cancer, as well as for drug delivery [7,8,9]. In particular, TiO_2_ photocatalysis is used to destroy hazardous compounds in water or air [2,10] through a process that require low energy, operates at ambient conditions and is able to mineralize organic pollutants using only atmospheric oxygen as the additional chemical species [11]. Unlike the bulk counterpart, nanosized TiO_2_ demonstrated improved performance, thanks to its high surface-to-volume ratio that greatly increases the density of active surface sites available for adsorption and catalysis. In addition, the size-dependent band gap of nanosized semiconductors allows to adjust the redox potentials of photogenerated electron–hole pairs to selectively control the photochemical reactions. Therefore, the reduced dimensions of the nanocatalyst allow the photo-generated charges to reach the catalyst surface, thus reducing the probability of undesired bulk recombination [4,12,13]. As a drawback, large bandgap semiconductors like TiO_2_ (3.2 eV for Anatase) respond only to UV light, thus resulting in low efficiency for the visible spectrum. The band gap excitation of semiconductor causes charge separation followed by the scavenging of electrons and holes by surface adsorbed species [14]. Visible-light-driven photocatalytic processes can thus be realized by doping TiO_2_ with non-metal [15,16,17,18], noble metal [19,20,21] or reduced and defect TiO_2_ engineering [22] etc., which represent different methods widely employed to narrow the wide band gap of TiO_2_. By controlling the surface treatment and medium conditions, it is possible to fine-tune photocatalytic properties of TiO_2_ to desired applications [14]. It is also well-known that, due to large curvature, TiO_2_ particles with sizes smaller than 20 nm have under-coordinated surface structure with square pyramidal geometry instead of an octahedral one [7,23]. Therefore, Ti atoms surface are very reactive, leading to the formation of charge transfer (CT) complexes with a red absorption shift, due to their binding with electron-donating ligands. The visible light activation of TiO_2_ has been observed upon the surface modification of colloidal TiO_2_ with L (+)-Ascorbic Acid (AA) [2,24,25]. AA, known as Vitamin C, is an important natural compound in the biology and chemistry fields, and particular interest is tuned towards its complex with metals. Complexes of AA with titanium (IV) should be relative strong and not prone to the metal catalyzed ligand oxidation that renders many metal ascorbate complexes so reactive [26]. The surface modifiers tend to enhance the surface coverage of the pollutant molecules on TiO_2_, inhibit the recombination process by separating the charge pairs, and extend the wavelength response [24]. It was found that compounds such as AA modify the surface of particles through the formation of π-π donor-acceptor complexes [2,24,27,28].

Our previous studies regarded the preparation of Polypropylene (PP) coated with only TiO_2_, TiO_2_ in combination with graphene and with gold nanoparticles and their application in the visible light photodegradation of Alizarin Red S (1,2-dihydroxy-9,10-anthraquinonesulfonic acid sodium salt or ARS) obtaining highly efficient dye degradation with an easy separation of the photocatalyst from the solution [29,30,31,32]. ARS is a widely used synthetic water soluble dye considered a refractory pollutant because of the difficulty in removing it through general treatments [29,30,31,32].

In this study, we want to continue our efforts regarding the extension of TiO_2_ light absorption into the visible range. For this purpose, AA modified TiO_2_ NPs supported on PP were prepared and investigated for the first time as visible light photocatalysts in water. We focused on three main points: first in the establishing a procedure for the preparation of specific new paste of AA modified TiO_2_ nanoparticles (NPs), second, to use this to obtain supported photocatalyst of defined qualities and third, to demonstrate the increasing photocatalytic ability in the degradation of ARS as target pollutants under visible light irradiation. This new photocatalyst showed a change in the adsorption mechanism with respect to that of pure TiO_2_ NPs and an improvement of the photocatalytic efficiency. The main advantages of the present approach were the easy preparation of the photocatalyst together with the use of the green compound AA. A comprehensive and in-depth characterization of the obtained photocatalyst permitted us to understand the reasons and types of surface modifications and the correlation between all the results.

## 2. Materials and Methods

### 2.1. Photocatalyst Preparation

Different types of new modified TiO_2_ pastes, named [AA-TiO_2_]_A_ were prepared by the addition of 6 g of Titanium (IV) dioxide Anatase nano-powdered (<25 nm), into 10 mL of distilled water containing different amounts of AA from 0.5 to 3.4 wt %, acetyl acetone 10% *v*/*v* and few drops of Triton X-100 with continuous grinding for 3 min. All the used chemicals were Sigma Aldrich products (Sigma Aldrich, St. Louis, MO, USA). Five different heterogeneous photocatalysts, named [PP@AA-TiO_2_]_A_, and containing different amount wt % of AA were prepared through the dip-coating technique on 20 cm^2^ surface of Polypropylene (PP) strips (3 M 2500 material). After the preparation, the photocatalyst was thermally dried in the oven at 110 °C.

### 2.2. Photocatalyst Characterization

A morphological study on the modified [AA-TiO_2_]_A_ and pure [TiO_2_]_A_ photocatalyst was made using Field Emission Scanning Electron Microscopy (FE-SEM, Sigma Family, Zeiss, Oberkochen, Germany) operated at 5–7 KV. All samples have been carefully prepared by removing the TiO_2_ paste from the plastic support material without a change of its properties. Then the obtained powder was deposited on aluminum stabs using self-adhesive carbon conductive tabs. The samples were sputtered with chromium (5 nm) by Quorum QT150 (Quorum, Laughton, UK) to prevent charging during the analysis. To study the structural variations of two compounds, pure [TiO_2_]_A_ and modified [AA-TiO_2_]_A_ were removed from the PP support and characterized by using the X-ray diffraction (XRD) technique. A customized horizontal Debye-Scherrer diffractometer was used for XRD measurements; this instrument is equipped with an INEL CPS 180 (INEL, Artenay, France) curved position sensitive detector in order to reduce drastically the acquisition time for each pattern. In order to optimize the efficiency this detector is filled with a Kr/CO_2_ gas mixture while, the absence of moving parts eliminates the need for mechanical scanning devices such as complex scanning goniometers used in conventional XRD instruments. A Mo K-alpha (lambda = 0.7093 Å) X-ray source is used generated by a Philips sealed X-ray tube and monochromatized through a graphite crystal along the 002 plane. The samples were positioned on the beam into glass capillaries (diameter 100 microns).

X-ray photoelectron spectroscopy (XPS) analysis has been obtained by means of an unmonochromatized X-ray source (Al Kα) and CLAM IV hemispherical spectrometer (VG Scientific Ltd., East Grinstead, UK) a constant passing energy (50 eV) for an overall lower than 1 eV half width at half-maximum (HWHM). The Raman analysis was performed using a micro-Raman spectrometer iHR320 (Horiba, Kyoto, Japan) in which the photocatalysts were excited with a green laser emitting at λ = 532 nm, at room temperature and the objective outlet was 100×. The photoluminescence (PL) measurements were achieved using a Perkin Elmer LS 45 luminescence spectrometer (Perkin Elmer, Waltham, MA, USA) equipped with a pulsed Xe flash lamp and, in particular, the PL spectra were collected at room temperature using an excitation wavelength of 290 nm in the range from 300–900 nm. The Diffuse Reflectance Spectra (DRS) were collected using an UV-Vis Spectrometer Lambda35 (Perkin Elmer, Waltham, MA, USA) with an integration sphere (P/N C6951014) in a range of wavelength from 200–1100 nm.

### 2.3. Adsorption and Photodegradation Processes

The adsorption isotherms of [PP@AA-TiO_2_]_A_ in the ARS adsorption under dark condition were analyzed by using four different concentration of ARS from 2.92 × 10^−5^ to 7.30 × 10^−5^ mol L^−1^.

The photocatalytic performance of [PP@AA-TiO_2_]_A_ photocatalyst was evaluated in the degradation of ARS 5.843 × 10^−5^ mol L^−1^ by using nine equal strips of [PP@AA-TiO_2_]_A_ inserted in a typical thermostated photoreactor system [29] connected with Cary 8454 Diode Array System spectrophotometer (Agilent Technologies, Santa Clara, CA, USA) with a continuous flux quartz cuvette (178.710-QS, light path 10 mm, Hellma Analytics, Müllheim, Germany) allowing a real-time analysis; the photoreactor was irradiated with visible light by using a tubular lamp (100 W, 1800 Lumen, LYVIA, (Arteleta International S.p.A., Milano, Italy); the spectral features are reported in Figure S1. All the spectrophotometric data were collected monitoring the decrease of ARS absorbance at fixed wavelength of 424 nm. The adsorption kinetics was evaluated in the same way under dark conditions.

## 3. Results and Discussion

### 3.1. Morphological and Structure Characterization

**Figure 1:**
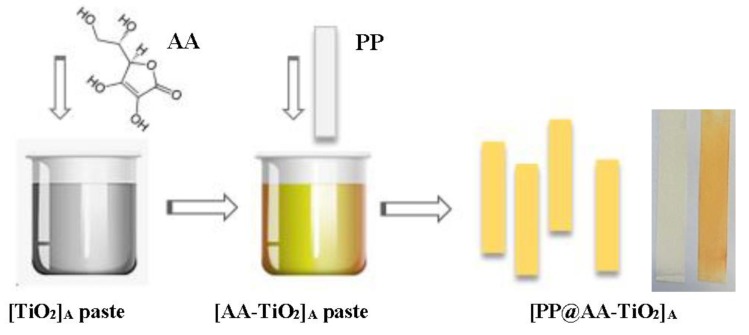
Schematic representation of the operative steps for the preparation of the new modified heterogeneous [PP@AA-TiO_2_]_A_ photocatalyst and photograph of [PP@AA-TiO_2_]_A_ prepared with two different AA amount wt %.

The overall procedure of the photocatalyst is schematically presented in Figure 1 where the addition of AA in the TiO_2_ pastes preparation, give the formation of yellow-brown color paste of [AA-TiO_2_]_A_ at pH equal to 5 with an increase color intensity as a function of AA amount wt %.

**Figure 2:**
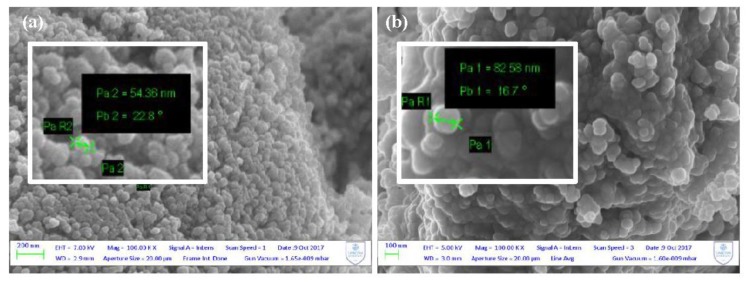
Scanning electron microscope (SEM) micrographs of (**a**) pure [TiO_2_]_A_; (**b**) modified [AA-TiO_2_]_A_ containing 2.5 wt % of AA.

SEM analysis was performed on the photocatalyst removed from the PP support; Figure 2 shows the SEM micrographs of pure [TiO_2_]_A_ and modified [AA-TiO_2_]_A_ samples at the same magnification revealing that the presence of AA as a surface modifier changes the morphological aspect of [AA-TiO_2_]_A_ photocatalyst.

In particular, the images reveal that the particle sizes change as a consequence of the addition of AA to TiO_2_ from around 55 nm for the pure [TiO_2_]_A_ photocatalyst (Figure 2a) to around 80 nm for the modified [AA-TiO_2_]_A_ (Figure 2b).

**Figure 3:**
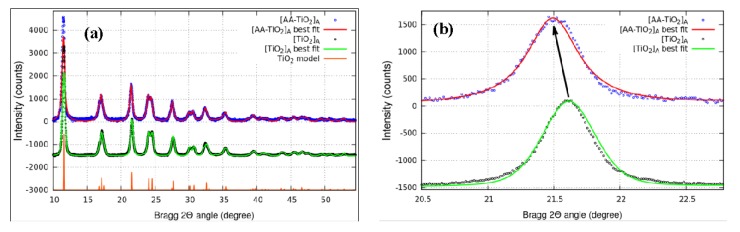
X-ray diffraction (XRD) patterns of (**a**) [TiO_2_]_A_ and [AA-TiO_2_]_A_ containing 2.5 wt % of AA; (**b**) Magnification of the 004 peak in the range in the range 20.5–23.0 deg.

To investigate the effect of AA as a surface modifier, the structural features of pure [TiO_2_]_A_ were characterized by XRD measurements and compared to that of [AA-TiO_2_]_A_ samples. Figure 3a showed the diffraction patterns of pure [TiO_2_]_A_ (black circle) and the modified [AA-TiO_2_]_A_ samples containing 2.5 wt % of AA (blue circle), the unit cell refinement (red and green lines), and the theoretical pattern of TiO_2_-anatase (orange line). From the analysis of these spectra, it is clearly visible that both samples exhibited a series of well-defined diffraction peaks attributable to the Anatase TiO_2_ crystal structure and no extra peaks have been observed in the XRD patterns. The values for the structural parameters of the cells obtained by the data refinement are shown in the Table 1. We found a close agreement between TiO_2_ [33] and [TiO_2_]_A_ values, while there is evidence of a parameter cell expansion of the [AA-TiO_2_]_A_ especially along the c-axis: this effect is clearly visible in Figure 3b where the data, in the range of 20–23 deg, show the shift of the 004 reflection directly connected with the vertical axis. It is known that the pH has an important role on the change of average crystallite size and the results obtained at about five pH derive from an increase of the average crystallite size as a consequence of the tensile strain [33]. The obtained results may be attributed to the lattice expansion with consequent incorporation of AA inside the crystalline lattice of the semiconductor material.

**Figure 4:**
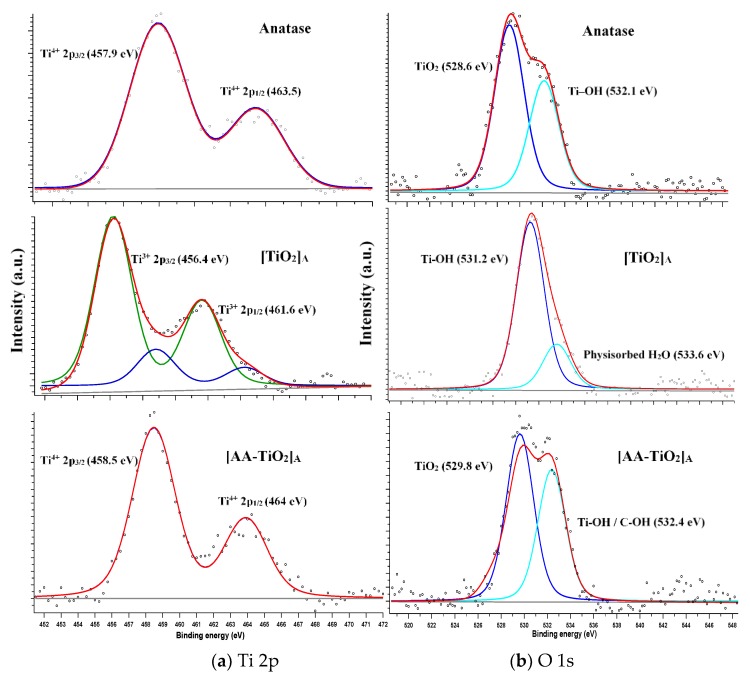
X-ray photoelectron spectroscopy (XPS) spectra of commercial Anatase (top), pure [TiO_2_]_A_ (in the middle) and modified [AA-TiO_2_]_A_ (bottom) containing 2.5 wt % of AA for (**a**) Ti 2p and (**b**) for O 1s.

The high resolution XPS spectra shown in Figure 4 for commercial Anatase, pure [TiO_2_]_A_ paste and modified [AA-TiO_2_]_A_ samples were realized in order to analyze the surface modification due to the presence of AA through the formation of the bidentate binuclear binding-bridging of AA-TiO_2_. All the obtained spectra were calibrated to the C 1s electron peak at 284.6 eV. Figure 4a,b show the peaks deconvolution of XPS spectra for Ti 2p and O 1s respectively. In Figure 4a (top), for commercial Anatase, two binding energy peaks at 458 and 463.5 eV are observed and are assigned to the Ti^4+^ 2p_3/2_ and 2p_1/2_ core levels, respectively [34,35,36,37]. Figure 4a (in the middle) for the pure [TiO_2_]_A_ heterogeneous photocatalyst shows additional strong peaks at 456.2 and 461.6 eV that are attributed to the Ti^3+^ 2p_3/2_ and 2p_1/2_ respectively formed on the TiO_2_ surface [38]. These peaks derived from a change of the TiO_2_ surface because of the paste preparation, in which the presence of water and acetylacetone obtain a partial complexation of monomeric Ti precursor [39] and the concomitant presence of Ti–OH. Figure 4a (bottom) shows strong binding energy peaks at 458.4 and 464 eV that are ascribed to the 2p_3/2_ and 2p_1/2_ core levels of Ti^4+^ and assigned to the chemical interaction between TiO_2_ and AA molecules [34,35,36,37]. The O 1s spectrum of commercial Anatase showed in Figure 4b (top) present two binding energy peaks at 528.6 and 532.1 eV that are attributed to Ti–O bond of lattice oxygen of TiO_2_ and non-lattice oxygen respectively as the Ti–OH terminal groups [40,41]. The pure [TiO_2_]_A_ sample is shown in Figure 4 (b, in the middle) in which two binding energy peaks at 531.2 and 533.6 eV are visible due to the presence of the OH group with oxygen at the bridging oxygen site (Ti-OH_b_) [42] and to the physiosorbed H_2_O which is present as a consequence of the influence of water molecules on the sample surface due to the paste preparation [41,42]. Figure 4b (bottom) for the modified [AA-TiO_2_]_A_ sample shows strong binding energy peaks at 529.8 and 532.4 eV that are assigned to the Ti–O surface bulk oxide lattice of TiO_2_ and OH as a terminal group with oxygen attached to the five-coordinated Ti^4+^ with an O–Ti^4+^ covalent bond [42] or C–OH due to the interaction of TiO_2_ with the AA [43]. It is known that almost 40% of the TiO_2_ surface consist of Ti atoms with incomplete coordination [24] that are four-fold coordinated to oxygen with two unfilled orbitals; consequently, they can accept two lone pair from electron donors to complete the octahedral coordination. The most possible conformation that leads to chelate ring structure that also offers higher stability derived by AA binding as a bidentate ligand through the two ene-diolate oxygen atoms with the function of the electron donor [27]. The five member AA ring structure is favorable for the Ti surface atoms, showing little distortion of bond angles and distances, while no evidence shows the involvement of glycolic side chain in the complex formation [27].

Raman spectra collected at room temperature did not show significant change with respect to the same in the absence of AA (Figure S2), the Raman active modes typical of TiO_2_ anatase remain unchanged, only a broadened band at 2800 cm^−1^ is detected that can be attributed to the fluorescence of AA. These results are in accordance also with the same sample modified by AuNPs, evidencing that AA not influences the crystallinity, crystallite size, and defects of TiO_2_ [32].

**Figure 5:**
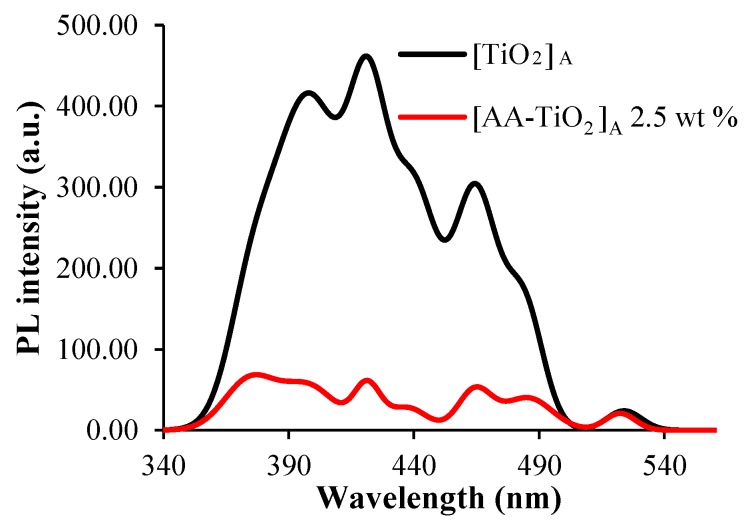
Photoluminescence (PL) spectra of [TiO_2_]_A_ (black line) and [AA-TiO_2_]_A_ with AA amount of 2.5 wt % (red line) excited at 290 nm in the wavelength range of 300–900 nm.

PL spectra (Figure 5) for pure [TiO_2_]_A_ and modified [AA-TiO_2_]_A_ sample with AA amount of 2.5 wt %, were also achieved in order to monitor the electron-hole pair recombination in response to the photon irradiation occurring on TiO_2_ surface mediated by the presence of AA as a surface modifier.

The PL spectra shows the peaks attributed to TiO_2_ [32], which decrease in intensity in the case of modified [AA-TiO_2_]_A_, indicated that the recombination process has been suppressed, resulting in higher photocatalytic activity. A de-convoluted PL emission spectra of both samples were reported in Figure S3a,b respectively.

### 3.2. Optical Characterization

**Figure 6:**
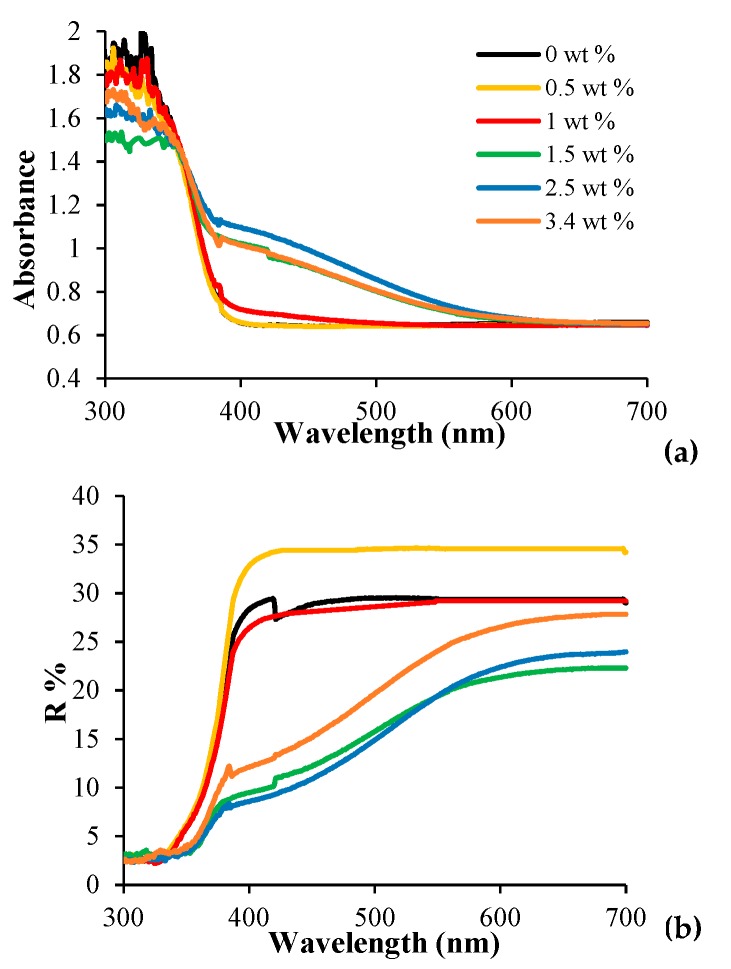

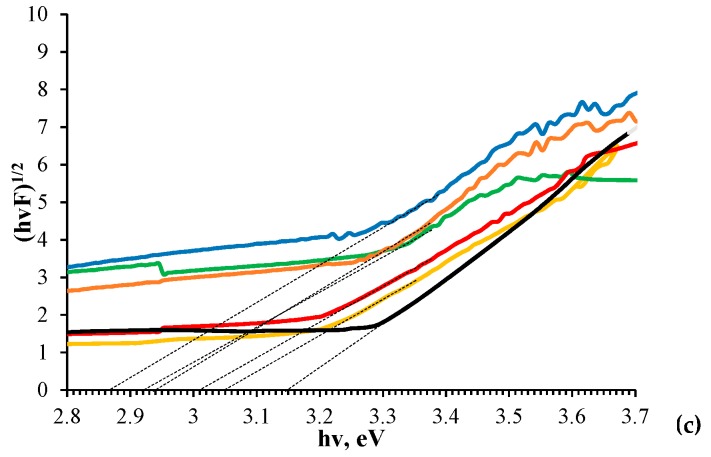
(**a**) UV-Vis Light Diffuse Reflectance spectra (DRS); (**b**) DRS spectra; (**c**) $E_{g}$ values, calculated with Kubelka–Munk method of [PP@AA-TiO_2_]_A_ photocatalysts containing different AA from 0 to 3.4 wt %.

Figure 6a shows the UV-Vis spectral change due to different AA amounts wt % of [PP@AA-TiO_2_]_A_ photocatalysts, demonstrating an increase in the absorption in the range of 370–570 nm. The appearance of a yellow-brown color on the modified [AA-TiO_2_]_A_ paste can be explained as a result of an intense ligand to metal charge transfer (LMCT) transition [27]; this is also clearly evidenced in the DRS spectra reported in Figure 6b that shows the change of optical properties of modified [PP@AA-TiO_2_]_A_ as a function of the AA amount. 

The DRS spectrum of pure [PP@TiO_2_]_A_ presents a sharp adsorption edge around 390 nm attributed to the electron’s excitation from the VB to CB (band gap 3.2 for Anatase [27] while AA does not absorb any light above 300 nm [27]). Generally, activated samples show a shift of absorption peak in the visible part of spectrum and, in particular, contain an extended absorption edge above 400 nm and a broad absorption peak between 550 and 900 nm [44]. The formation of a Ti^IV^-AA surface complex results from a change of the absorption threshold [27] of the modified [PP@AA-TiO_2_]_A_ photocatalyst that shifted towards the visible part of the spectrum. In fact, in this case, the modified sample presents a long tail extending up to ca. 600 nm as a consequence of the charge transfer complex formation between Ti^IV^ atoms. AA introduces electronic states that are spread across the band gap, resulting in a diffused absorption spectrum [24]. These results confirm the formation of the AA-TiO_2_ charge-transfer complex into TiO_2_ paste that could narrow the energy band gap ($E_{g}$) of the modified [PP@AA-TiO_2_]_A_ photocatalyst. $E_{g}$ values of pure and modified samples were calculated by applying the Kubelka-Munk method [32] by the linear fit of the curves of Figure 6c, where F represents the Kubelka-Munk function, obtaining the respective values (Table 2) by the intercept in the x-axis.

The results of Table 2 clearly show that the surface modification with AA positively influences the $E_{g}$, improving the photocatalytic activity. In fact, the $E_{g}$ value is around 3.15 eV for [PP@TiO_2_]_A_ [32], while the minimum $E_{g}$ value of 2.87 eV is obtained for [PP@AA-TiO_2_]_A_ in the presence of AA 2.5 wt %.

### 3.3. Equilibrium and Kinetic Studies of ARS Adsorption

**Figure 7:**
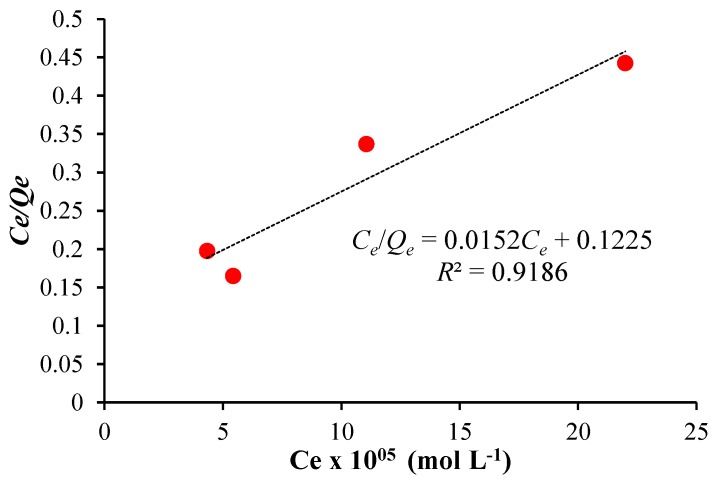
Langmuir isotherm graph for ARS adsorption on [PP@AA-TiO_2_]_A_.

In order to study the presence of surface modifiers and how they influence ARS adsorption on [PP@AA-TiO_2_]_A_ photocatalyst, the adsorption data has been analyzed by the application of the adsorption isotherm models of Freundlich and Langmuir [29]. While ARS adsorption on [PP@TiO_2_]_A_ photocatalyst occurred according to the Freundlich isotherm model [29], instead the results obtained with [PP@AA-TiO_2_]_A_ (Figure 7) showed that the dye adsorption fitted well the Langmuir isotherm model $C_{e}/Q_{e}=1/K_{L}+a_{L}C_{e}/K_{L}$, where $C_{e}$ is the ARS solution concentration (mol L^−1^), $Q_{e}$ is the adsorbed ARS amount at equilibrium (mol L^−1^). The results showed Langmuir constants $K_{L}$ and $a_{L}$ of 8.16 and 1.24 × 10^5^, respectively, a theoretical saturation capacity of the TiO_2_ surface $Q_{0}$ of 6.56 × 10^−5^ indicating therefore a change in the adsorption mechanism due to the presence of AA. [PP@AA-TiO_2_]_A_ permitted therefore the adsorption of AA molecules in monolayer mode and with the same adsorption energy.

### 3.4. Visible Light Photoactivity of [PP@AA-TiO_2_]_A_

ARS adsorption under dark condition and photodegradation under visible light were monitored by the decrease of the ARS absorption spectra at 424 nm in acidic conditions.

**Figure 8:**
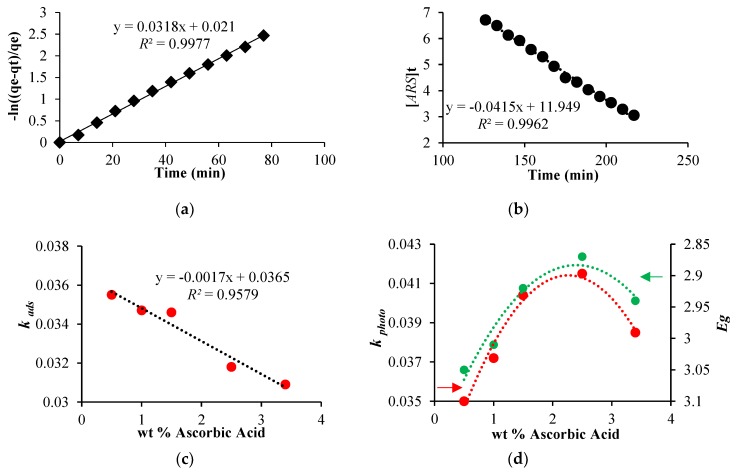
(**a**) Pseudo first order adsorption kinetic; (**b**) zero order photodegradation kinetic by using [PP@AA-TiO_2_]_A_ containing 2.5 wt %; (**c**) correlation between $k_{ads}$ and AA wt %; (**d**) correlations between the $k_{photo}$ and $E_{g}$ vs. AA wt %.

As reported in Figure 8a, the adsorption of ARS on the modified [PP@AA-TiO_2_]_A_ photocatalyst follows a pseudo first order kinetic in which the adsorption kinetic constant $k_{ads}$ is expressed by the equation $\ln[\left(q_{e}-q_{t}\right)]/q_{e}=k_{ads}t$, where $q_{t}$ and $q_{e}$ are the amount of adsorbed dye at time *t* and its equilibrium concentration, respectively [29,30,31,32].

In addition, in the presence of [PP@AA-TiO_2_]_A_ the photodegradation rate becomes proportional to the ARS concentration during time, in accordance with zero order kinetic, ${\left[ARS\right]}_{t}=-k_{photo}t$ [45], where $k_{photo}$ is the photodegradation kinetic constant, while [*ARS*]*_t_* is ARS concentration at time *t*. In particular, in this case, the simultaneous presence of two components as AA and ARS onto TiO_2_, influenced the kinetic order of the photodegradation process with respect to that of first-order kinetics with only TiO_2_, probably due to AA distribution on the TiO_2_ surface. Table 2 reports the results obtained for $k_{ads}$ and $k_{photo}$ values relative to the process of ARS 5.84 × 10^−5^ mol L^−1^ on [PP@AA-TiO_2_]_A_ prepared with different AA wt %, while in Figure 8 shows the linear graphs about the kinetics of adsorption (Figure 8a) and photodegradation (Figure 8b).

According to Table 2, as shown in Figure 8c, a correlation among the $k_{ads}$ and the AA amount used for the preparation of the modified photocatalyst is observed demonstrating that by increasing the concentration of AA in the TiO_2_ paste, a decrease of the $k_{ads}$ was found. In addition, an increase of the concentration of AA, corresponds to a decrease of $E_{g}$ and an increase of $k_{photo}$ until the sample with AA concentration of 2.5 wt % after which both show a reverse trend (Figure 8d).

**Figure 9:**
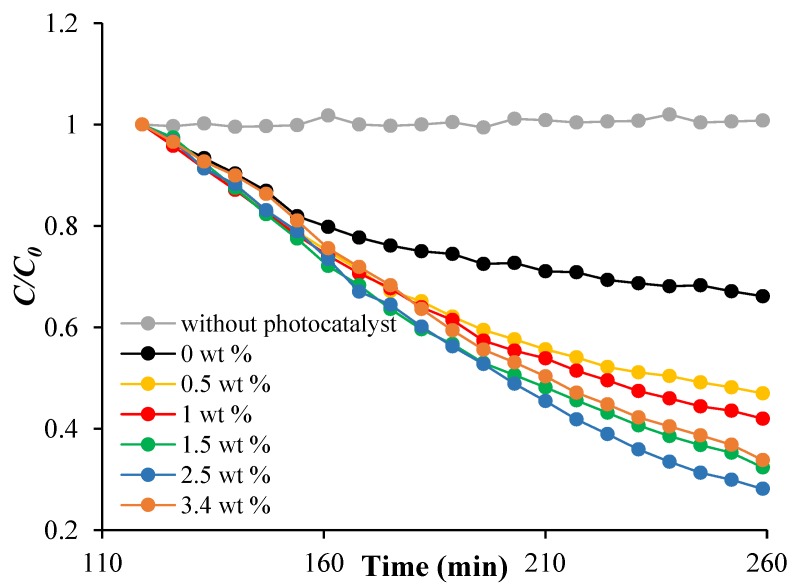
ARS photodegradation versus time for [PP@-TiO_2_]_A_, different [PP@AA-TiO_2_]_A_ photocatalysts and without photocatalyst under visible light.

In Figure 9 is reported the ARS photodegradation time for different [PP@AA-TiO_2_]_A_, [PP@-TiO_2_]_A_ photocatalysts, and without photocatalyst under visible light, these results show the positive effects of the presence of AA; in particular the best condition is obtained with AA 2.5 wt %.

**Figure 10:**
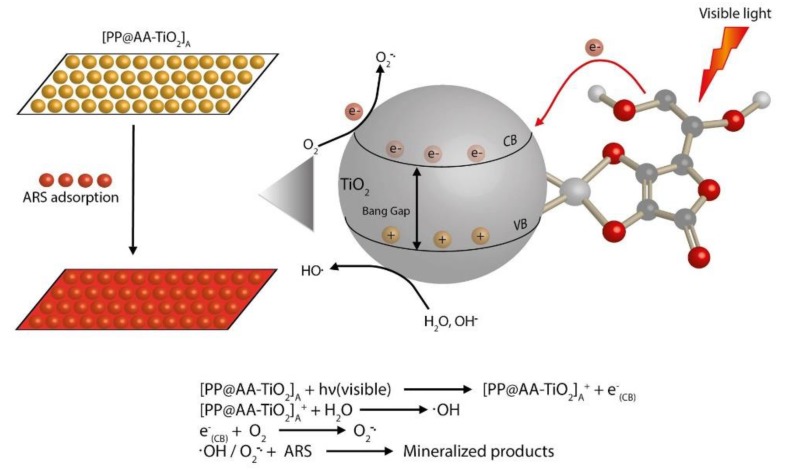
Mechanism of photocatalytic process by using [PP@AA-TiO_2_]_A_ for the degradation of ARS solution.

The mechanism of the photocatalytic process under visible light of the modified [PP@AA-TiO_2_]_A_ photocatalyst can be resumed as reported in Figure 10. When [PP@AA-TiO_2_]_A_ is irradiated by visible light, an electron transfer from the AA to CB semiconductor occurred and superoxide molecular ions are formed by the presence of molecular oxygen. Then, the formed radicals drive the photocatalytic degradation of ARS adsorbed on a modified photocatalyst [46].

## 4. Conclusions

A [PP@AA-TiO_2_]_A_ yellow-brown photocatalyst with high visible-light photocatalytic activity in the ARS dye degradation was successfully and for the first time realized by cover PP material with a specific TiO_2_ paste modified with AA.

The new modified photocatalyst has been widely characterized by using SEM, XRD, XPS, PL and Raman techniques. SEM images reveal that the particle sizes changed as a consequence of the addition of AA to TiO_2_, XRD measurements demonstrate a lattice expansion with consequent incorporation of AA inside the crystalline lattice of TiO_2_ material, while XPS measurements showed a superficial change of Ti oxidation state that change from Ti(III) to Ti(IV).

In addition, due to the interaction of AA with TiO_2_, a lower PL emission intensity has been obtained, demonstrating a lower charge recombination that enhances the photo-produced electron transition to AA with an improved electron-hole separation.

The experimentally calculated $E_{g}$ values, by using [PP@AA-TiO_2_]_A_ photocatalysts, decreased with the increase of surface modifier concentration according to the increase of performances. The best $E_{g}$ value of 2.87 eV obtained with 2.5 wt % of AA corresponds to a $k_{photo}$ of 0.0415. The obtained results have demonstrated that this new photocatalyst has proved to be 2.08 times more effective of only TiO_2_ prepared in absence of AA.

In addition, a change in the adsorption mechanism with respect to that of pure [PP@TiO_2_]_A_ has been observed, while kinetic studies on the photocatalytic performance of [PP@AA-TiO_2_]_A_ in the visible light photodegradation of ARS showed an improvement of the photocatalytic efficiency that is strictly correlated with $E_{g}$ values.

## Figures and Tables

**Table 1:** Parameter cell values for TiO_2_ [33], [TiO_2_]_A_ and [AA-TiO_2_]_A_.

Sample	a (Å)	c (Å)
TiO_2_ [33]	3.785	9.514
[TiO_2_]_A_	3.787	9.526
[AA-TiO_2_]_A_	3.808	9.565

**Table 2:** $E_{g}$ , $k_{ads}$ and $k_{photo}$ values for [PP@AA-TiO_2_]_A_ containing different AA wt %.

AA wt %	$E_{g}\;(\mathrm{eV})$	$10^{2}\;k_{ads}\;(\min^{-1})$	$10^{2}\;k_{photo}\;(\min^{-1})$
0	3.15	3.75	1.99
0.5	3.05	3.55	3.50
1	3.01	3.47	3.72
1.5	2.92	3.46	4.04
2.5	2.87	3.18	4.15
3.4	2.94	3.09	3.85

## Supplementary Materials

The following are available online at http://www.mdpi.com/2079-4991/8/8/599/s1, Figure S1: Raman spectra of [PP@TiO_2_]_A_ and [PP@AA-TiO_2_]_A_ containing 2.5 wt % of AA. Figure S2. Raman spectra of [PP@TiO_2_]_A_ and [PP@AA-TiO_2_]_A_ containing 2.5 wt % of AA. Figure S3. Gaussian fitted PL spectra of [TiO_2_]_A_ (a) and [AA-TiO_2_]_A_ (b), dotted line: fitting of deconvolution study.
