# Supplementary material for: Band Gap Implications on Nano-TiO2 Surface Modification with Ascorbic Acid for Visible Light-Active Polypropylene Coated Photocatalyst
Source: Nanomaterials (Basel). 2018 Aug 7;8(8):599. doi: 10.3390/nano8080599 (PMC6116251; doi:10.3390/nano8080599)
Supplement: Supplementary file 1 [file nanomaterials-08-00599-s001.pdf]

Supplementary materials:

Band gap implications on nano-TiO_2 surface modification with ascorbic acid for visible light-active polypropylene coated photocatalyst

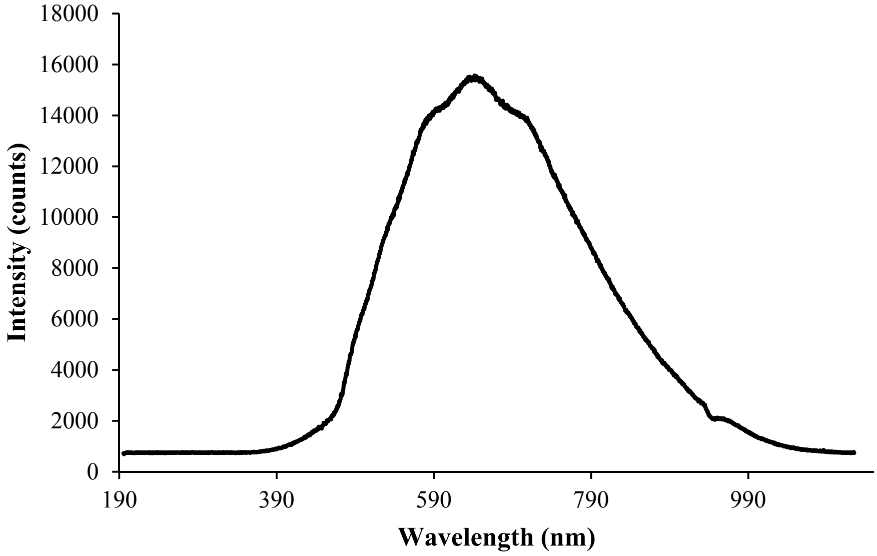

Figure S1. Emission spectrum of tubular lamp (100W, 1800 Lumen, LYVIA).

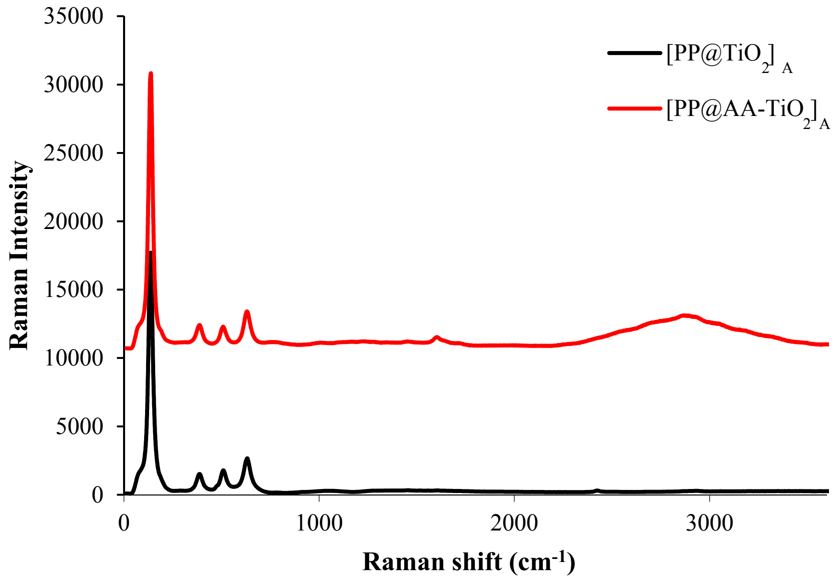

Figure S2. Raman spectra of $[PP@TiO_2]_A$ and $[PP@AA\text{-}TiO_2]_A$ containing 2.5 wt % of AA.

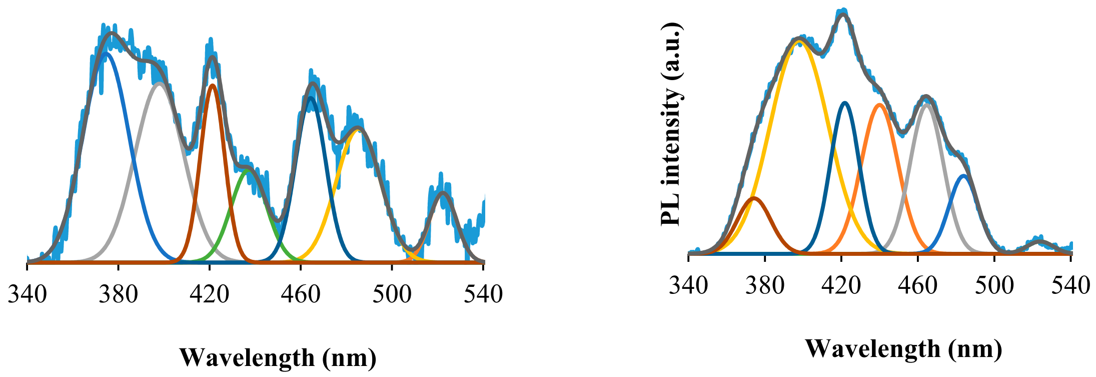

(a) $[TiO_2]_A$ **(b)** $[AA\text{-}TiO_2]_A$

Figure S3. Gaussian fitted PL spectra of $[TiO_2]_A$ (**a**) and $[AA\text{-}TiO_2]_A$ (**b**), dotted line: fitting of deconvolution study.
